# Liquid Biopsy Potential Biomarkers in Prostate Cancer

**DOI:** 10.3390/diagnostics8040068

**Published:** 2018-09-21

**Authors:** Jochen Neuhaus, Bo Yang

**Affiliations:** Department of Urology, Research Laboratory, University Leipzig, D-04103 Leipzig, Germany; zp_yangb@sumhs.edu.cn

**Keywords:** prostate cancer (PCa), biomarkers, liquid biopsy, diagnosis high-grade PCa, risk stratification, risk calculators

## Abstract

Prostate cancer (PCa) is the second most common cancer in men worldwide with an incidence of 14.8% and a mortality of 6.6%. Shortcomings in comprehensive medical check-ups in low- and middle-income countries lead to delayed detection of PCa and are causative of high numbers of advanced PCa cases at first diagnosis. The performance of available biomarkers is still insufficient and limited applicability, including logistical and financial burdens, impedes comprehensive implementation into health care systems. There is broad agreement on the need of new biomarkers to improve (i) early detection of PCa, (ii) risk stratification, (iii) prognosis, and (iv) treatment monitoring. This review focuses on liquid biopsy tests distinguishing high-grade significant (Gleason score (GS) ≥ 7) from low-grade indolent PCa. Available biomarkers still lack performance in risk stratification of biopsy naïve patients. However, biomarkers with highly negative predictive values may help to reduce unnecessary biopsies. Risk calculators using integrative scoring systems clearly improve decision-making for invasive prostate biopsy. Emerging biomarkers have the potential to substitute PSA and improve the overall performance of risk calculators. Until then, PSA should be used and may be replaced whenever enough evidence has accumulated for better performance of a new biomarker.

## 1. Introduction

Prostate cancer (PCa) is the second most common cancer in men worldwide, with an incidence of 14.8% (estimated at 1.1 million in 2012) and a mortality of still 6.6% (an estimated 307,000 deaths in 2012), which is at 5th rank among cancers. The five-year prevalence of 148.6/100,000 is the highest seen in all cancers in adult men [1].

There is a significant variation of incidence, with the highest rates seen in Western and Northern Europe, Australia/New Zealand, and Northern America and the lowest rates in Asian countries, which may be partly due to the comprehensive use of prostate-specific antigen (PSA) testing and close monitoring of the male population in more developed regions. As recently reported in China, the poor coverage of PSA monitoring seems to be the major cause of advanced PCa at first diagnosis, translating into a high mortality of 44% in China compared to 23% in the EU and 14% in the USA [2,3,4].

In this review we will evaluate the performance of available and emerging biomarkers in risk stratification, focusing on liquid biopsies and distinguishing high-risk Gleason score (GS) ≥ 7 tumours from indolent, low-risk tumours, especially in biopsy-naïve patients.

## 2. Current PCa Diagnostics

At present, the recommendations of the American Urological Association (AUA) and the European Association of Urology (EAU) guidelines for the clinical examination methods of prostate cancer include digital rectal examination (DRE), rectal ultrasound (TRUS) examination, and prostate biopsy [5,6,7]. 

Biopsy is still the only standard in preoperative diagnosis. This invasive operation causes obvious physical pain to patients and the detection rate was closely related to the number of puncture points and prostate volume, and is likely to cause haematuria, urinary retention or infection, sepsis, and other serious complications leading to distress. At the same time, DRE and TRUS have disadvantages such as poor accuracy and are affected by the skill and experience of the operator and other subjective factors. For instance, 24% of 126 patients with suspected DRE had no PCa on biopsies in a prospective study by Leyten and co-workers [8].

In addition, all three, DRE, TRUS and biopsy, are inevitably faced with an important issue, namely low sensitivity to early cancer, after which the patient has missed the most ideal treatment time [9,10,11].

As proposed by Dr TA Stamey in the early 1980s, PSA diagnostics rapidly developed into comprehensive screening programs in the USA and worldwide [12]. PSA population screening in 6260 Americans showed a significant decrease of high- and intermediate-risk patients from 68.9% to 52.3% and an increase of low-risk disease from 31.2% to 47.7% between 1989 and 2002 [13]. PSA screening caused prostate cancer mortality to fall by 21%, demonstrated in the 13-year follow-up of the European Randomised Study of Screening for Prostate Cancer (ERSPC), indicating that early diagnosis and treatment of prostate cancer were indeed significantly improved by the popularity of serum PSA screening [14,15,16]. 

However, in the past decade more and more evidence accumulated challenging the benefit of extensive PCa screening programs. Comprehensive PSA screening, in particular, may lead to significant overdiagnosis and overtreatment [17,18,19]. Overdiagnosis and unnecessary prostate biopsies were calculated to occur in 23–42% in PSA screening programs [20]. Thus, there are considerable concerns about the predictive value of PSA levels, PSA density, and even first biopsy in the group of patients eligible for active surveillance following the recommendations of the EAU [21]. 

One major problem of PSA screening is the different cutoff values used. The threshold of 4.0 ng/mL (commonly used with the Tandem-R, Hybritech test) has to be revised based on the findings of 15.2% PCa in men with PSA ≤ 4 ng/mL in a cohort of 2950 men (aged 62–91 years) by Thompson and co-workers [22]. A considerable 14.9% of those were diagnosed with high-grade (GS ≥ 7) tumours. Even at very low PSA levels of ≤0.5 ng/mL, they found 6.6% PCa, of which 12.5% were high-grade tumours [22]. These findings clearly illustrate the diagnostic shortcomings of PSA. 

Age-adjusted PSA cutoffs were tested to improve performance. While age-adjusted PSA cutoffs for total serum PSA (tPSA) and complexed serum PSA (cPSA) were superior to a fixed cutoff in a cohort of 3597 men who underwent routine biopsy, they could not improve the PCa detection rate of approximately 39% within the range of 2.0 ng/mL–20.0 ng/mL [23].

In light of those diagnostic restrictions, there is still an ongoing dispute on PSA testing worldwide. In the USA, PSA screening was evaluated by the U.S. Preventive Services Task Force (USPSTF), leading to amendment of the recommendations on PSA screening in 2008 [24]. The American Urological Association (AUA) stated that PSA screening should apply only to men aged 55 to 69 years and suggested that routine examination be performed once every two years or longer [7,18,25]. 

However, this diagnostic practice may significantly increase the risk of missing PCa in men <55 years [26] and might increase mortality, which has to be analysed after longer follow-up [27,28]. Nevertheless, recent data analysis shows a continuous increase in the morbidity of biopsies in conjunction with reduction of total biopsies since the 2008 USPSTF recommendations [29]. Amongst the many attempts to solve this diagnostic dilemma, only a few biomarkers are established in clinical practice.

In summary, to date no single serum or urine biomarker or biomarker panel meets the requirements for highly sensitive and specific detection of PCa and differentiation between indolent and significant PCa. Imaging technologies have been greatly improved but still are not sufficiently validated or standardized. Some of the diagnostic tools are already established, such as PSA and its derivatives; others are under critical evaluation and some are exploring the potency of the latest high-end analytics to improve (i) early detection of PCa, (ii) risk stratification, (iii) disease prognosis, and (iv) treatment monitoring.

## 3. Current PCa Biomarker Tests for Discrimination-Significant and Indolent PCa

The need for better PCa diagnostics has led to a huge number of new strategies for meaningful combinations of established and innovative approaches to open up new biomarker resources. Table 1 gives an overview of commercially available biomarker tests, using liquid biopsy for the detection of high-grade (≥GS 7) PCa and assisting the physician with identifying patients for prostate biopsy and those eligible for active surveillance (for detailed information and more specialized tests, see [30]). Table 1 also demonstrates that PSA cutoff values vary between studies. Age-adjusted use of PSA cutoff values could significantly improve the sensitivity of PSA testing [23].

### 3.1. Prostate-Specific Antigen (PSA)

PSA, alone or in combination with free/total PSA (f/t PSA) ratio, formerly thought to be of value for distinguishing PCa from BPH, shows only limited sensitivity and insufficient specificity (Table 1; recent meta-analysis by Huang et al. [31]).

Longitudinal PSA screening to determine PSA velocity has been initially described to distinguish PCa from BPH in men aged >60 years at an average rate of change (ng/mL per year) of ≥0.75 with 90% specificity compared to 60% single PSA value ≥4 ng/mL [32]. However, in the following studies these promising results could not be confirmed [31].

### 3.2. Prostate Health Index and Derivates

The Prostate Health Index (PHI Beckman Coulter, Atlanta, GA, USA) was FDA-approved in 2012. The PHI score combines total, free, and [–2] proPSA in one test and a score is calculated indicating the probability of PCa positive biopsy (phi-score = [−2] proPSA/fPSA) × √tPSA) [46]. 

In an early meta-analysis, AUCs of %[–2] proPSA and PHI were comparable in patients with PSA values of 2–10 ng/mL, reaching between 0.76 and 0.78 for prediction of PCa-positive biopsies [47]. As recently analysed by White and co-workers, PHI alone impacts the decision-making of physicians and resulted in a significant reduction in biopsies of 40% [48]. However, the PHI score was not very good at predicting high-grade PCa and as such did not help with clinical decision-making in a large study using pT3 stage and/or GS ≥ 7 as outcome measures [49] (see also Table 1 [35]).

### 3.3. 4KScore^®^ Test

Promising data have also been reported for the 4KScore^®^ Test (OPKO Lab, Nashville, TN, USA) using 4-Kallikrein markers in blood serum after DRE. In a recent study including 496 participants with PSA ≥ 3.0 ng/mL, the accuracy of predicting PCa GS ≥ 7 was AUC 0.738 in the standard model (PSA + age) and AUC 0.820 in the advanced model integrating 4KScore^®^ Test (*p* < 0.001). In a model with 6% cutoff, the risk calculated by age and 4KScore^®^ would avoid 43% of biopsies, detect 119 of 133 (89.5%) GS ≥ 7 high-risk tumours, and delay diagnosis of 14 (10.5%) of the significant tumours [36].

### 3.4. Progensa™ (Gen-Probe Inc., San Diego, CA, USA)

The prostate cancer gene 3 (PCA3) test detects long non-coding RNA (lncRNA), which has been shown to be associated with PCa. The Progensa™ PCA3-score calculates the ratio of PCA3 and PSA mRNA of exosomes isolated from post-DRE urine and has been proposed for the identification of patients eligible for active surveillance [50]. It was FDA-approved in 2012 and several studies reported variable performance (sensitivity: 58–78%; specificity: 57–72%) for detection of PCa (Table 1) [39,40,41]. The NPV was reported to be 88% and 90%, respectively, which was regarded as helpful for biopsy decision-making [40,41]. However, as pointed out by Vickers and co-workers, PCA3 was approved by the FDA only to add the decision of repeat biopsy. In biopsy-naïve patients, there is a high risk of missing high-grade PCa with low levels of PCA3 [51].

### 3.5. Further Non-Commercial and Integrative Tests

Several other tests are already available for risk evaluation in patients with elevated PSA, usually at a threshold of ≥4 ng/mL (Table 1). While their positive predictive value is low (28–36%), those tests showed highly negative predictive values (88–98%), which make them valuable for clinical decision-making on invasive biopsy diagnostics. Tomlins et al. reported avoidance of 35–47% of biopsies using the MiPS test (University of Michigan, MLabs) [43]. Van Neste calculated that 42% of total biopsies and 53% of unnecessary biopsies can be avoided by combining the SelectMDx (MDx Health, Irvine, CA, USA) measuring HOXC6 mRNA and DLX1 mRNA in post-DRE urine with serum PSA, PSA density, DRE status, age, and family history [44].

As summarized in Table 1, more or less critical restrictions regarding the target patients apply to most biomarker test available. Only a few are useful in a broad clinical setting, as required for screening or routine check-up examinations, i.e., for application in biopsy-naïve patients. Especially for the detection of critical high-risk patients (“risk of Gleason score ≥ 7”) who need to undergo prostate biopsy, the specificity of the available tests is poor. For detailed information, the reader is referred to the literature given in Table 1.

## 4. Do We Need More Biomarkers, or Do We Need a New, Consistent Concept?

In view of the huge number of different biomarkers available and new approaches, one has to ask whether there is a realistic chance that these advanced methods will finally provide a set of biomarkers able to meet all requirements in PCa detection, stratification, and monitoring. Most probably, we will need a combination of specialized biomarkers with good performance within their restricted fields. The primary goal should be to reduce the need for invasive prostate biopsies to improve the benefit‒harm balance.

An ideal biomarker concept should support low-invasive, organ-saving treatments if possible; radical surgery if necessary at the earliest time point to avoid PCa progression to metastatic and androgen-insensitive disease [52].

## 5. Emerging Biomarkers for Detection of Significant PCa

### 5.1. Polypeptides

Seminal plasma is a body fluid directly related to the prostate. Therefore, PCa specific analytes are expected to be available at higher concentrations and better accessible than in urine or blood. An in-depth proteome analysis of expressed prostatic secretions (EPS) was conducted in 2010 by Drake and co-workers to provide a resource for the development of biomarkers [53]. In a small cohort of patients with advanced (*n* = 8) or organ-confined (*n* = 8) prostate cancer, a total of 624 unique proteins were identified in EPS by mass spectrometry [54]. Fourteen candidates with 133 differently expressed proteins were further analysed for suitability as biomarkers, including PSA and PAP, which were significantly elevated in organ-confined PCa. The authors concluded that EPS-urines are a promising source for new PCa biomarkers [54].

In a multicentre, open-label case/control study our group analysed 125 patients for PCa-specific polypeptides in seminal plasma from fresh ejaculate donation after physiological liquefaction. The idea was to create stable conditions reflecting the enzymatic activity of pathological protease network in PCa, and to analyse the resulting protein fragments, ≤20 kDa polypeptides by capillary-electrophoresis mass spectroscopy (CE-MS). We were able to define a panel of 11 polypeptides from seminal plasma-based CE-MS analysis with 80% sensitivity at 82% specificity in discriminating patients with GS 7 and organ-confined (<pT3a) or advanced disease (≥pT3a) [55].

Proteomic signatures of polypeptides have also been used to detect PCa in the urine of biopsy-naïve patients without known PCa or suspect DRE [56]. The biomarker panel of 12 polypeptides detected PCa with 89% sensitivity and 51% specificity (AUC 0.70). By inclusion of age and fPSA, the performance was augmented to 91% sensitivity and 69% specificity [56]. Unfortunately, the data were not analysed for prediction in high-risk patients.

### 5.2. Metabolites

Metabolites were shown to closely reflect aggressiveness of PCa [57]. A prospective study including 1122 cases tested the performance of sarcosine to predict the risk of prostate cancer. This study revealed an association of serum sarcosine levels normalized to alanine with low-grade (non-aggressive) PCa but no association with aggressive PCa [58], and a recent study showed that sarcosine is not indicative of PCa in urine [59].

Post-DRE urine pellet is used as a source for metabolites to predict high-grade PCa (GS ≥ 7) in the Polarix^®^ test (Metabolon Inc., Morrisville, NC, USA). In a retrospective study, McDunn and co-workers identified metabolites associated with the aggressiveness of a tumour and constructed a panel of four metabolites (5,6-dihydrouracil, choline phosphate, glycerol, and methylpalmitate) predicting the probability of organ-confined PCa with an accuracy of AUC = 0.62. Using a panel of three metabolites (7-a-hydroxy-3-oxo-4-cholestenoate, pregnen-diol disulfate, and mannosyl tryptophan), they were also able to improve the prediction of progression-free survival to AUC = 0.64 [57]. While these results are promising, the performance of urine metabolites is still not satisfying. However, the study provides the basis for further development of metabolite biomarkers.

Most interestingly, PCa-specific metabolites have been found in urine exosomes, implying potential use as a new biomarker source to address PCa pathogenesis and progression. Out of 248 metabolites identified, 76 were differentially expressed in PCa and BPH [60]. 

Metabolomics is a hot topic in current biomarker research. However, so far, even large studies did not successfully identify meaningful metabolites [61].

### 5.3. MicroRNA (miRNA) 

MiRNAs have been acknowledged to be important for gene regulation in normal and pathological conditions. Based on tissue analyses, dozens of miRNAs have been shown to be dysregulated in PCa (see [62,63]).

In a comprehensive screening study using radical prostatectomy samples of 34 patients, 34 miRNA were significantly upregulated in the tumour epithelium compared to normal epithelium [64]. The authors also compared GS 6 PCa with high-grade GS ≥ 8 PCa tissue. They found 18 differentially expressed miRNAs (*p* < 0.005): 11 were up- and seven were downregulated (Table 2).

Schaefer et al. reported five upregulated and 10 downregulated miRNAs during miRNA microarray analysis of 76 radical prostatectomy specimens comparing matched tumour and adjacent normal tissues [65]. The expression of five miRNAs correlated with Gleason score, and upregulated miR-96 predicted biochemical recurrence (Table 2). 

However, Stephan et al.’s study of miR-183 (upregulated) and miR-205 (downregulated) failed to detect high-grade PCa in patients with and without PCa (38 each group) using urine sediment, while PCA3 was able to separate those patient groups [66]. In a recent meta-analysis, Song et al. identified from an extensive literature survey 10 upregulated and 14 downregulated miRNAs with potential for separating PCa from BPH or normal controls (Table 2). Furthermore, high expression of miR-32 and let-7c differentiated local from metastatic PCa. The authors also found that the expression profiles of urine, blood serum, and tissue differed considerably [62].

Circulating miRNAs were isolated from various body fluids, including blood plasma and serum being protected against ribonuclease degradation by inclusion in lipid compartments, extracellular vesicles of 40–5000 nm in diameter [67].

In serum, miR-141 levels can distinguish PCa from healthy controls with an AUC of 0.907 with 60% sensitivity at 100% specificity in a cohort of 25 PCa and 25 age-matched healthy control individuals [68]. In a recent study Porzycki et al. found that the combination of miR-141, miR-21, and miR-375 could distinguish PCa (mean PSA of 21.3 ng/mL) from healthy controls with an AUC of 0.864 and a sensitivity of 93% at 63% specificity. However, the group sizes were quite small (20 PCa vs. eight healthy controls), requiring further validation of the findings in larger cohorts [69].

Recently, Tinay et al. found a significant upregulation of miR-9-3p, miR-330-3p-3p, and miR-345-5p in PCa patients (*n* = 25) compared to healthy controls (*n* = 20). MiR-345-5p was further analysed due to its direct targeting of CDKN1A encoding the cyclin-dependent kinase inhibitor p21 [70]. Interestingly, the overlap between the miRNAs in serum with tissue miRNAs is limited to miR-21, miR-141, and miR-375 (boldface and labelled “l” in Table 2).

In urine, PCa-specific miRNAs patterns can be detected in exosomes by next-generation sequencing (NGS) and RT-qPCR. For example, miR-196a-5p and miR-501-3p levels were significantly downregulated in a preliminary study of 28 PCa (GS ≥ 7) vs. 19 healthy controls [71]. In a larger study, including 215 PCa patients, 23 BPH patients, and 62 asymptomatic control individuals, Stuopelyte et al. found 100 out of 754 miRNAs scanned deregulated in PCa. MiR-148a and miR-375 were the most abundant miRNAs in urine and showed high sensitivity and specificity (85.31% and 65.22%, respectively), with an AUC of 0.79 in differentiation between PCa (*n* = 72) and BPH (*n* = 23). In combination with serum PSA, the AUC was 0.85, with 84.29% sensitivity at 76.19% specificity. Within the grey zone PSA levels of 4–10 ng/mL AUC increased to 0.90 with 83.87% sensitivity at 81.82% sensitivity [72]. Overlapping miRNAs were in boldface and labelled “m” in Table 2.

None of the current miRNA approaches provide high PPV for the detection of high-grade PCa. The largest NPV of 0.939 has been reported to predict the absence of high-grade PCa compared to BPH for a 14-miRNA panel: miR-24, -26b, -30c, -93, -100, -103, -106a, -107, -130b, -223, -146a, -451, -874, and let-7a [73].

The high number of miRNAs found to be dysregulated in PCa and the ability of subsets to either detect PCa, differentiate high-grade from low-grade PCa, or predict recurrence-free/overall survival encourages further attempts to define miRNA biomarker panels. Most interestingly, the overlap between tissue and liquid biopsies is rather limited. This problem has to be investigated more deeply.

### 5.4. Gene Expression of PCa-Related Genes in Exosomes

Exosomes can be used to measure gene expression of PCa-related genes (among others: SPDEF, ERG and PCA3). Combined into a score (ExoDx Prostate IntelliScore urine exosome assay (Exosome Diagnostics, Inc., Waltham, MA, USA)) with standard of care parameters: prostate-specific antigen level, age, race, and family history [42], this score was able to predict high-risk PCa, defined as GS ≥ 7, with a sensitivity of 92%. However, specificity was low (34%) resulting in a positive predictive value (PPV) of only 36%. In contrast, the negative predicted value (NPV) was high (91%), thereby unnecessary biopsies could have been avoided in 27%, missing only 5% of patients with high-risk PCa (GS 4 + 3) [42], indicating a considerable clinical value of tests providing high NPV, even if the PPV is low (Table 1).

### 5.5. Long Non-Coding RNA (lncRNA)

Non-coding RNA makes up the vast majority of our genetic information. Only <3% of the human DNA comprises protein-coding gene sequences. LncRNAs are key regulators of the genome and their involvement in several disease states, especially cancer has recently emerged [74,75,76]. Thus, lncRNAs are regarded as promising biomarkers as well as therapeutic targets in urologic cancers [77,78]. Several biomarker tests including lncRNA (PCA3) are already on the market (Table 1).

Recently investigated promising lncRNAs include: TINCR [79], FR0348383 [80], SChLAP1 [81], and MALAT1 [82] (Table 3). LncRNA biomarker research is rapidly expanding and more lncRNA biomarkers are likely to emerge in the near future [83,84].

### 5.6. Circulating Tumour Cells (CTC)

At the moment circulating tumour cells (CTC) are not used for early detection of high-grade PCa because they are rarely detected in localized PCa [89,90,91,92]. CTCs are being investigated for use as a prognostic biomarker of mCRPC and to predict treatment efficacy [93,94,95,96]. However, the first long-term follow-up studies have questioned the prognostic value of preoperative CTCs for the prediction of early biochemical recurrence. Meyer et al. detected CTCs in only 11% (17/152) of patients before radical prostatectomy. The CTC counts did not correlate with PSA levels, disease status, or biochemical recurrence [97]. Recently, Murray et al. concluded that the biological characteristics of circulating prostate cells (CPCs) may be more important than the number of circulating cells. They found that patients with CD82-negative CPCs had a worse prognosis in a study of 285 men at a follow-up of 10 years. CD82 is a tumour suppressor and the expression on CPCs may indicate high metastatic potential [98].

## 6. Integrative Scoring Systems/Risk Calculators

The overall goal of all biomarkers is the improvement of the prediction of the individual risk of the patient. To this end, standard null hypothesis significance testing (NHST) methods are not conducive, since they do not report quantitative percent individual risk evaluation. Bayesian data analysis can overcome this weakness and provide direct access to meaningful risk evaluation. Risk calculators such as the online Prostate Cancer Prevention Trial Risk Calculator (PCPTRC) developed in 2006 predicting the likelihood of detecting no versus low-grade (GS < 7) versus high-grade (GS ≥ 7) in a biopsy for an individual patient can be continuously adjusted on the basis of newly available epidemiologic data [99,100,101]. New biomarkers replacing lower-performing ones may be included when available, thus continuously improving the performance of those calculators. Several complex scoring models for risk assessment of PCa GS ≥ 7 have been developed, including population adaption: the Stockholm model 3 (STHLM3) [45], the Rotterdam Prostate Cancer Risk Calculator (RPCRC) [102], the Indonesian prostate cancer risk calculator (IPCRC) [103], a Chinese (Hong Kong) adaptation of the ERSPC risk calculator [104], the Huashan risk calculator [105], and the Chinese Prostate Cancer Consortium Risk Calculator (CPCC-RC) [106].

## 7. Conclusions

**Figure 1:**
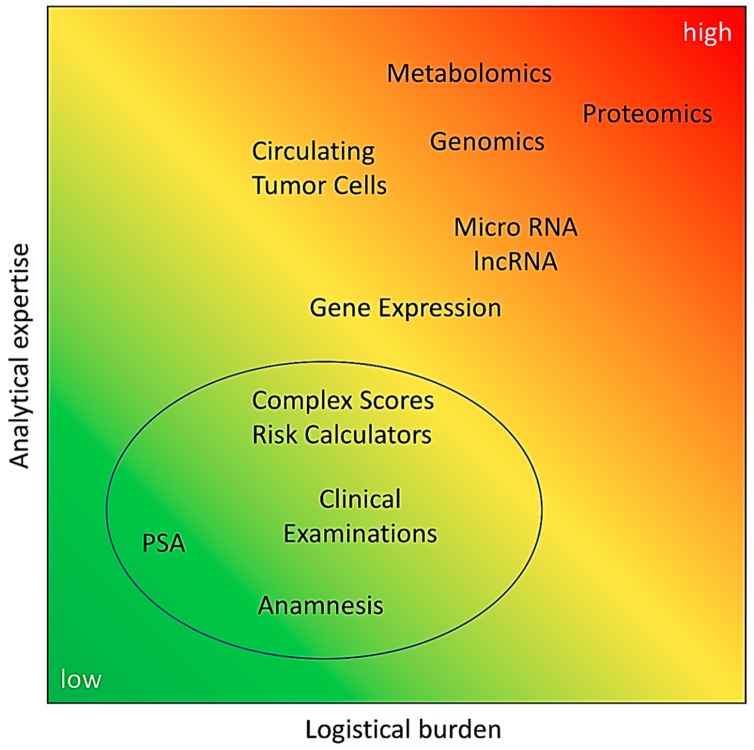
Feasibility of liquid biomarker-based diagnostics. The financial burden is coded by colour (green = low, yellow = medium, red = high); at present, advanced analytical methods come with higher high technical requirements and the need for very high analytical expertise; generally, gene expression, genomics, proteomics, and metabolomics require specialized analysis laboratories. In most cases, e.g., for untargeted analyses, standards have not been defined yet; this accelerates the threshold for comprehensive establishment in the clinical routine. For instance, proteomic analyses are still expensive, require high analytical expertise, and are not comprehensively available. On the other hand, proteomic analyses are fairly good and standardized. Comparing metabolic analyses, they require higher expertise than the more standardized proteomics, but are less expensive. The logistical burden grows with the complexity of the clinical and analytical requirements; circle indicates currently well established methods of PCa diagnostics.

In this review we focused on the liquid biopsy biomarkers currently in use and emerging for distinguishing patients with low, insignificant PCa from patients with high-risk PCa with a Gleason score ≥ 7. Biomarker development faces some common challenges that may limit the usability of biomarkers in clinical routine. The accuracy of transrectal ultrasound (TRUS)-guided needle biopsy is limited by a false negative rate of 23% [107]. Serial biopsies can improve the detection rate of organ-confined PCa from 77% at first biopsy to 99% at fourth biopsy [107]. However, since this initial study in 2002 the number of biopsy cores to be obtained increased from quadrant biopsy (four cores) to sextant biopsies (six cores) and, recently, to a standard of 10–12 cores, as recommended by the guidelines of the EAU. Nevertheless, detection rates are still in the range of 35% [36]. This results in significant uncertainty when using systematic biopsy as a reference standard. In addition, there is a significant upgrading of tumour grade of up to 56.7%, as demonstrated by studies comparing biopsy and final Gleason score after radical prostatectomy [21,108,109]. This causes another serious problem in defining the reference standard in biomarker studies, because whole-gland histopathological evaluation is only available after tumour radical prostatectomy and in rare cases of prostate enucleation due to large volume BPH. Furthermore, in healthy control groups neither biopsy material nor whole-gland tissue is available, reducing the determination of “tumour-free” status to clinical observation and exclusion of other biomarkers (in practice, mostly suspicious PSA levels). Another challenge is the population bias, e.g., shown in metabolomic studies. Special care has to be taken in conception, sampling, and sample processing to account for ethnic and lifestyle differences [110,111]. Population-based adjustment of biomarker panels and cutoffs is required, e.g., for Asian and Western countries [112,113,114,115]. All biomarkers have to compete against PSA and most outperform PSA in certain patient groups. While PSA assays are standardized, comparable, and easy to handle, with a low logistical burden, many of the novel biomarkers make higher demands on clinical staff, organization, laboratory equipment, and data analysis (Figure 1).

In addition, the superiority to PSA has to be validated in large prospective studies, which usually takes at least five years. There are already good data for the biomarkers established in the market (Table 1), but only a few of the novel biomarkers can provide clinical data. Furthermore, distinct restrictions apply to the tests (“targeted patients” in Table 1), which need to be taken into account when comparing the performance of different biomarkers. Currently, histopathological evaluation of needle biopsies is the gold standard and the basis of treatment decisions. Biomarkers should be able to predict the initial biopsy outcome in respect of high-grade disease, i.e., they should have a highly positive predictive value. At present, none of the available biomarkers and tests alone can achieve this goal.

Therefore, because of the low application threshold, PSA monitoring is indispensable at the moment, and should be integrated into routine health examinations of men aged ≥45 years, as recommended in the latest 2018 German S3-guidelines for Prostate Cancer [116]. In case of suspect PSA findings, additional biomarkers should be used to further characterize disease state and safe stratification of patients into treatment groups. Risk calculators should be used for transparent decision-making and to improve the inclusion of the patient.

## Figures and Tables

**Table 1:** PCa biomarker tests for prediction of high-grade PCa (GS ≥ 7).

Biomarker(s)	Source	Commercial Product	Predict	Avoid Biopsies	Sens.	Spec.	AUC	PPV	NPV	Targeted Patients	Ref.
PSA	blood (serum)	**Tandem-R^®^** monoclonal immunoradiometric assay (Hybritech Inc., San Diego, CA, USA)	PCa on first biopsy	n.a.	79% at PSA ≥ 4 ng/mL	59% at PSA ≥ 4 ng/mL	0.64	40%	89%	age > 50 years PSA ≥ 4 ng/mL	[33]
PSA	blood (serum)	**Tandem-R^®^** (Hybritech)	PCa (vs. BPH/Controls)	n.a.	78% at PSA ≥ 4 ng/mL	60% (PCa vs. BPH); 94% (PCa vs. Control at PSA ≥ 4 ng/mL	n.r.	n.r.	n.r.	age > 60 years	[32]
PSA velocity (0.75 ng/mL/year)	blood (serum)	**Tandem-R^®^** (Hybritech)	PCa (vs. BPH/Controls)	n.a.	72% at PSA ≥ 4 ng/mL	90% (PCa vs. BPH); 100% PCa vs. Control: at PSA ≥ 4 ng/mL	n.r.	n.r.	n.r.	age >60 years	[32]
PSA	blood (serum)	**Access Hybritech^®^**	Risk of GS ≥ 7	n.a.	90% at PSA ≥4.3 ng/mL	9% at PSA ≥ 4.4 ng/mL	0.55	n.r.	n.r.	age ≥ 50 years PSA 4–10 ng/mL, neg. DRE	[34]
fPSA/tPSA	blood (serum)	n.r.	PCa (vs. BPH)	n.a.	70% (pooled data)	58% (pooled data)	0.76 (pooled data)	41%	86% ^(1)^	PSA 4.0–10.0 ng/mL	meta-analysis [31]
PHI (p2PSA/fPSA × √tPSA)	blood (post-DRE serum)	**PHI**, prostate health index Beckman Coulter, Atlanta, GA, USA)	Risk of GS ≥ 7	n.r.	90% (pooled data)	17% (pooled data)	0.67 (pooled data)	n.r.	n.r.	age ≥ 50 years PSA 4–10 ng/mL, neg. DRE	meta-analysis [35]
p2PSA/fPSA (%p2PSA)		**PHI**, prostate health index Beckman Coulter, Atlanta, GA, USA)	Risk of GS ≥ 7	n.r.	96% (pooled data)	9% (pooled data)	0.54 (pooled data)	n.r.	n.r.	age ≥ 50 years PSA 4–10 ng/mL, neg. DRE	meta-analysis [35]
PHI (p2PSA/ fPSA × √tPSA)	blood (post-DRE serum)	**PHI**, prostate health index Beckman Coulter, Atlanta, GA, USA)	Risk of GS ≥ 7	30.1%	90% (cutoff 29.8)	30% (cutoff 29.8)	0.71	n.r.	n.r.	age ≥ 50 years PSA 4–10 ng/mL, neg. DRE	[34]
intact PSA, free PSA, total PSA, kallikrein-related peptidase 2 (hK2)	blood (post-DRE serum)	**4KScore^®^** Test (OPKO Lab, Nashville, TN, USA)	Risk of GS ≥ 7	43%	n.r.	n.r.	0.82	n.r.	n.r.	PSA ≥ 3 ng/mL;	[36]
expression of 8 auto-antibodies against: CSNK2A2, cestrosomal protein 164 kDa, NK3 homeobox 1, aurora kinase interacting protein 1,5′-UTR BMI1, ARF6, chromosome 3′-UTR region Ropporin/RhoEGF, desmocollin 3	blood (serum)	**Apifiny^®^** (Armune Bioscience, Kalamazoo, MI, USA)	Risk of GS ≥ 7	n.r.	60% at PSA > 4 ng/mL [37]	69% at PSA > 4 ng/mL [37]	0.69 at PSA > 4 ng/mL [37]	30% [37]	89% [37]	PSA ≥ 2.5 ng/mL, initial biopsy	[37,38]
prostate cancer gene 3 (PCA3) + PSA mRNA ratio	post-DRE urine	**Progensa™** (Gen-Probe Inc., San Diego, CA, USA)	PCa	n.r.	58% [39] 78% [40] 76% [41]	72% [39] 57% [40] 52% [41]	0.68 [39] n.r. [40] 0.80 [41]	n.r. [39] 34% [40] n.r. [41]	n.r. [39] 90% [40] 88% [41]	age ≥ 50 years neg. prior biopsy, repeat biopsy	[39,40,41]
exosomes (EV) + (SOC: prostate-specific antigen level, age, race, family history); gene expression (targets revealed): SPDEF, ERG and PCA3	urine	**ExoDx^®^** Prostate IntelliScore urine exosome assay (Exosome Diagnostics, Inc., Waltham, MA, USA)	Risk of GS ≥ 7	n.r.	92%	34%	0.73	36%	91%	PSA 2–20 ng/mL, initial biopsy	[42]
serum PSA + urine PCA3 mRNA + urine TMPRSS2:ERG mRNA	blood (serum); post-DRE urine	**Progensa™** (Hologic, Bedford, MA, USA); **MiPS** test; University of Michigan (MLabs)	Risk of GS ≥ 7	35–47%	n.r.	n.r.	0.77 (PSA + T2:ERG + PCA3	n.r.	n.r.	elevated PSA (initial biopsy), prior negative biopsy (repeat biopsy)	[43]
HOXC6 mRNA + DLX1 mRNA + serum PSA + PSA density + DRE status + age + family history	post-DRE urine	**SelectMDx** (MDx Health, Irvine, CA, USA)	Risk of GS ≥ 7	42% of total; 53% of unne-cessary biopsies	91% (HOXC6 + DLX1)	36% (HOXC6 + DLX1)	0.76 (HOXC6 + DLX1); 0.90 + clin. Para-meters	28%	98%	PSA > 4 ng/mL; negative index biopsy	[44]
STHLM3 risk-based model: PSA, fPSA, iPSA, hK2, β-microseminoprotein (MSMB), macrophage inhibitory cytokine 1 (MIC1), genetic polymorphisms [232 SNPs], age, family history, previous prostate biopsy, DRE, prostate volume	blood	various	Risk of GS ≥ 7	32% biopsies (GS ≥ 7); 44% benign biopsies	n.r.	n.r.	n.r.	n.r.	n.r.	PSA ≥ 3 ng/mL; age 50–69 years; highly selected patients; validation in standard populations needed	[45]

Abbreviations: sensitivity (sens.); specificity (spec.); receiver-operation-characteristics (ROC) area under the curve (AUC); positive predictive value (PPV); negative predictive value (NPV); benign prostate hyperplasia (BPH); Gleason score (GS); free PSA (fPSA); total PSA (tPSA); not applicable (n.a.); not reported (n.r.). ^(1)^ calculated by authors from Table 1 in Huang et al., 2018 [31].

**Table 2:** Micro RNAs in prostate cancer diagnosis.

Reference	Song et al. 2018 [62]		Schaefer et al. 2010 [65]		Walter et al. 2013 [64]	
Type	Meta-Analysis of 104 Studies		Original Article		Original Article	
Samples	Tissue, Blood, Urine		RPE Frozen Tissue (76 PCa, 79 PCa)		FFPE RPE Tissue (37 PCa)	
Method(s)	Various		miRNA Microarray; 470 miRNAs		PCR Array Profiling	
Measure	Expression in PCa		Expression in PCa		Expr. in GS ≥ 8 vs. GS 6	
	miR-1 ↓	a	miR-16 ↓		miR-9 ↑	i
	miR-18a ↑	a	miR-31 ↓	j	miR-27 ↓	i
	**miR-21** ↑	**c,l**	miR-96 ↑	e,g,j	**miR-30c ↑**	**h,l**
	miR-23b ↓	a	miR-125b ↓	k	miR-34 ↑	i
	miR-27b ↓	a	miR-145 ↓		miR-92 ↓	i
	miR-30c ↓	a,c	miR-149 ↓	e	miR-96 ↓	i
	miR-31 ↑	b	miR-181b ↓		miR-122 ↑	h,i
	miR-34a ↑	a	miR-182 ↑	e	miR-125a ↑	h
	miR-99b ↓	a	miR-182 * ↑		miR-125 ↓	i
	miR-106b ↑	a	miR-183 ↑	f	miR-126 ↓	i
	miR-129 ↓	c	miR-184 ↓		miR-138 ↑	i
	miR-139-5p ↓	a	miR-205 ↓	e,f,j,k	miR-144 ↑	i
	**miR-141 ↑**	**a,l**	miR-221 ↓		miR-146b-5p ↑	h
	miR-145 ↓	c	miR-222 ↓	k	miR-148 ↓	i,m
	miR-152 ↓	a	**miR-375 ↑**	**e,l,m**	miR-181a ↑	h
	miR-182 ↑	a			miR-181c ↑	h
	miR-183 ↑	a			miR-184 ↑	h,i
	miR-187 ↓	a			miR-193 ↑	i
	miR-200a ↑	a			miR-193b ↑	h
	miR-200b ↑	a			miR-198 ↑	i
	miR-204 ↓	a			miR-214 ↑	h
	miR-205 ↓	a			miR-215 ↑	i
	miR-224 ↓	a			miR-222 ↓	i
	miR-301a ↑	a			miR-335 ↑	h,i
	**miR-375** ↑	**a,d,l,m**			miR-373 ↑	i
	miR-452 ↓	a				
	miR-505 ↓	a				
	let-7c ↓	a,b,c				

FFPE = formalin-fixed paraffin-embedded; TURP = transurethral resection of the prostate; RPE = radical prostatectomy; a = differentiate PCa from BPH/HC; b = differentiate advanced metastatic from local/primary PCa; c = prediction of poor recurrence free survival; d = worse overall survival; e = AUC of 0.88 combining 5 miRNAs; f = AUC of 0.88 combining two miRNAs; g = can predict biochemical recurrence; h = *p* < 0.005 in PCa vs. normal epithelium; i = differentiate GS ≥ 8 from GS 6; j = correlation with Gleason score; k = correlation with tumour stage; l = of diagnostic value in serum; m = of diagnostic value in urine; * = indicates reverse miRNA sequence; ↑ = upregulated; ↓ = downregulated.

**Table 3:** Long non-coding RNAs (lncRNA) potential biomarkers.

Name	Function	Diagnostic Value	Reference
PCA3 ↑ (prostate cancer associated 3)	increase of cell proliferation, migration and invasion; inhibition of apoptosis; [85]	predict risk of GS > 7	[86]
TINCR ↓ (Terminal differentiation induced non-coding RNA)	growth inhibition via TRIP13 suppression [79]	not determined	[79]
FR0348383 ↑	unknown	predict PCa-positive biopsy; avoid 52% unnecessary biopsies without missing high-grade PCa	[80]
SChLAP1 ↑ (SWI/SNF complex antagonist associated with prostate cancer 1)	increase of cell proliferation, metastasis via downregulation of miRNA-198 and activation of MAPK1 pathway [87]	predict high-risk, lethal PCa; biochemical recurrence after RPE	[81]
MALAT1 ↑ (metastasis-associated lung adenocarcinoma transcript 1)	interacts with EZH2, promoting proliferation and invasion [88]	predict PCa-positive biopsy; discriminate between PCa and BPH, PCa and HC	[82]

RPE = radical prostatectomy; HC = healthy controls; ↑ = upregulated; ↓ = downregulated.
